# Peripheral Dopamine 2-Receptor Antagonist Reverses Hypertension in a Chronic Intermittent Hypoxia Rat Model

**DOI:** 10.3390/ijms21144893

**Published:** 2020-07-10

**Authors:** Elena Olea, Inmaculada Docio, Miguel Quintero, Asunción Rocher, Ana Obeso, Ricardo Rigual, Angela Gomez-Niño

**Affiliations:** 1 Departamento de Enfermería, Universidad de Valladolid, 47005 Valladolid, Spain; olea@ibgm.uva.es; 2 Instituto de Biología y Genética Molecular, Consejo Superior de Investigaciones Científicas-Universidad de Valladolid, 47005 Valladolid, Spain; rocher@ibgm.uva.es (A.R.); aobeso@ibgm.uva.es (A.O.); rrigual@ibgm.uva.es (R.R.); 3 Departamento de Bioquímica, Biología Molecular y Fisiología, Universidad de Valladolid, 47005 Valladolid, Spain; inmadocio@hotmail.com (I.D.); migquico@hotmail.com (M.Q.); 4 Departamento de Biología Celular, Histología y Farmacología, Universidad de Valladolid, 47005 Valladolid, Spain

**Keywords:** chronic intermittent hypoxia, carotid body, hypertension, dopamine, domperidone, peripheral dopamine antagonist

## Abstract

The sleep apnea-hypopnea syndrome (SAHS) involves periods of intermittent hypoxia, experimentally reproduced by exposing animal models to oscillatory PO_2_ patterns. In both situations, chronic intermittent hypoxia (CIH) exposure produces carotid body (CB) hyperactivation generating an increased input to the brainstem which originates sympathetic hyperactivity, followed by hypertension that is abolished by CB denervation. CB has dopamine (DA) receptors in chemoreceptor cells acting as DA-2 autoreceptors. The aim was to check if blocking DA-2 receptors could decrease the CB hypersensitivity produced by CIH, minimizing CIH-related effects. Domperidone (DOM), a selective peripheral DA-2 receptor antagonist that does not cross the blood-brain barrier, was used to examine its effect on CIH (30 days) exposed rats. Arterial pressure, CB secretory activity and whole-body plethysmography were measured. DOM, acute or chronically administered during the last 15 days of CIH, reversed the hypertension produced by CIH, an analogous effect to that obtained with CB denervation. DOM marginally decreased blood pressure in control animals and did not affect hypoxic ventilatory response in control or CIH animals. No adverse effects were observed. DOM, used as gastrokinetic and antiemetic drug, could be a therapeutic opportunity for hypertension in SAHS patients’ resistant to standard treatments.

## 1. Introduction

The sleep apnea-hypopnea syndrome (SAHS) involves episodes of total or partial closure of the upper airway during sleep giving rise to periods of intermittent hypoxia, sleep fragmentation and inspiratory efforts. Current epidemiologic studies show that patients with SAHS have increased risk of hypertension, but the mechanisms are not well defined [1]. Experimental observations showed that animal models exposed to oscillatory PO_2_ mimicking intermittent hypoxia produced arterial hypertension and heart alterations [2,3,4] like those found in SAHS patients [5]. 

It is currently accepted that chronic intermittent hypoxia (CIH) produces carotid body (CB) sensitization generating an increased input to the brain stem which originates sympathetic hyperactivity, followed by hypertension. The CB is involved in the origin of hypertension and the functional elimination of CB after denervation or ablation prevents the development of hypertension [6,7,8,9,10]. The hyperactivity of the sympathetic system seems to be the link between CB and hypertension [11]. The CB is a dopaminergic organ in most species [12] but the functional meaning of dopamine (DA) is controversial, mainly due to the presence of DA receptors in the carotid sinus nerve endings, the efferent sensory nerve innervating the CB, and in the chemoreceptor cells. These DA autoreceptors can modulate chemoreceptor cells and therefore, the CB functionality and capability to respond to hypoxia. It is also known that the CB expresses high levels of dopamine D2 receptors (D2R) located in the carotid sinus nerve and in chemoreceptor cells [13,14] which are negatively coupled to adenyl cyclase and calcium channels, and activate inhibitory G-protein-activated inwardly rectifying potassium channels [15]. The CB synthesizes and releases DA from chemoreceptor cells in a highly correlated manner with the intensity and duration of hypoxia [12,16]. Exposure to CIH produces hypersensitization of carotid chemoreceptors and CIH increases DA content in CB from rat [16] and guinea pig [17], even if the increase of DA is much smaller than the induced by chronic sustained hypoxia [16,18].

The study was designed to test the hypothesis that blocking D2R on peripheral chemoreceptors could modify the CB hypersensitivity produced by chronic exposure to intermittent hypoxia and consequently, to minimize CIH related effects. Domperidone (DOM) is a selective peripheral dopaminergic D2R antagonist that only marginally crosses the blood brain barrier and therefore lacks central activity [19]. It has also been reported that, unlike other similar antagonists, DOM has no α2-adrenoreceptor blocking activity [20]. Therefore, DOM can be used to examine the effect of endogenous DA in peripheral chemoreceptor response or in other peripheral targets. We administered acute or chronic DOM to rats exposed to CIH and we found that DOM reversed the hypertension induced by CIH exposure. This observation could be due to a CIH CB desensitization, producing a similar effect to that obtained with CB denervation. No adverse effects were observed at the doses used. DOM is used as a gastro-esophageal motility regulator and antiemetic drug with no major side effects [21]. 

## 2. Results

### 2.1. Effect of Domperidone on CIH Induced Hypertension

**Figure 1:**
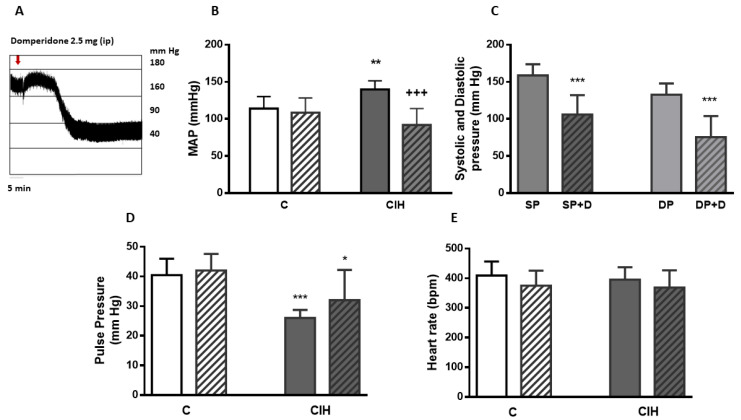
Acute effect of domperidone on arterial blood pressure. Effect of DOM (2mg/kg^−1^; ip) on **C** and CIH rats. (**A**) shows a single blood pressure recording from a CIH rat. (**B**) represents mean arterial blood pressure (MAP) from C animals (white bar) and the acute effect of DOM (striped bar), and MAP from CIH (gray bar) and the acute effect of DOM (gray striped bar). ** *p* < 0.01 vs. C; ^+++^ *p* < 0.001 vs. CIH. Data are represented as mean and SD from 6—9 animals; Two-way Anova with Sidak’s multiple comparisons test. (**C**) shows systolic and diastolic pressure from CIH animals before (SP and DP) and after administration of DOM (SP+D and DP+D); *** *p* < 0.001 SP vs. SP+D; ^+++^ *p* < 0.001 DP vs. DP + D represented as mean and SD from 9 rats. Two-way Anova with Sidak’s multiple comparisons test. In (**D**,**E**) pulse pressure and heart rate from the C and CIH before and after DOM administration (CD and CIHD) are shown. Data are mean and SD from 6–9 rats.

Mean arterial blood pressure (MAP) is the most common and easy cardiocirculatory parameter to establish CIH effects [2]. The acute effect of DOM was studied on rats divided into two groups: control (C) and exposed to CIH during 15 days, enough time to develop hypertension in rats [22,23]. The effect of 2 mg/kg^−1^ DOM intraperitoneal (ip) was assessed in both groups of animals. Figure 1A represents the acute effect of DOM on a single pressure recording from a CIH rat showing the hypotensive effect of DOM 30 minutes after ip injection. Figure 1B shows MAP from C and CIH animals and the acute effect of DOM in both groups of rats. In C animals DOM did not modified MAP values (114 ± 16 and 108 ± 19 mmHg). However, MAP significantly decreased from 140 ± 11 mmHg to 92 ± 22 mmHg (*** *p* < 0.001 vs. CIH) after ip administration of DOM in CIH rats. In part C of the figure the effect of the drug on systolic and diastolic pressure values from CIH animals is represented, showing the decrease of both: systolic pressure from 158 ± 15 to 106 ± 26 mm Hg (*** *p* < 0.001) and diastolic pressure from 132 ± 15 to 75 ± 28 mm Hg (*** *p* < 0.001) from 9 animals. No significant changes were observed in control animals. Figure 1D shows arterial pulse pressure value in both conditions, 40.4 ± 5.5 and 42.1 ± 5.8 mm Hg from C and CD and 26 ± 2.7 and 32.1 ± 10.2 mm Hg from CIH and CIHD, significantly different without DOM treatment (*** *p* < 0.001 CIH vs. C) and with DOM treatment (* *p* < 0.05 CIHD vs. CD). Figure 1E shows heart frequency, varying between 369 ± 58 and 409 ± 45 beats/min^−1^, also without statistical differences between C and CIH or after the injection of DOM in each condition.

**Figure 2:**
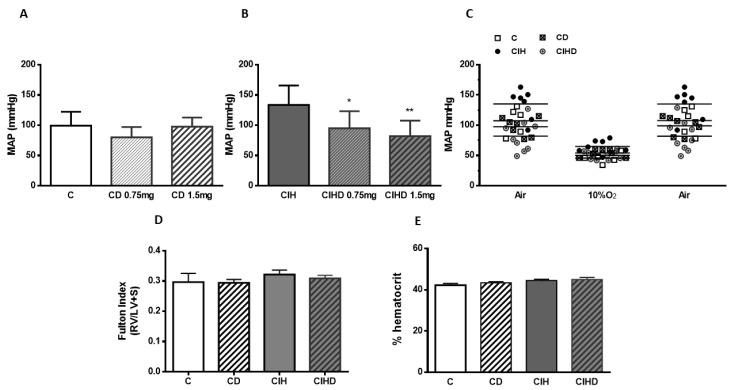
Chronic effect of domperidone on arterial blood pressure. In (**A**,**B**) chronic effect of DOM (0.75 or 1.5 mg/day; 15 days) on MAP from C (**A**) and CIH (**B**) rats. Data are mean and SD from 6—10 animals. * *p* < 0.05 and ** *p* < 0.01 vs. CIH; One-way ANOVA with Tukey’s multiple comparisons test. In (**C**) individual measures of MAP from the four group of animals (DOM 0.75 mg/day; 15 days) in basal (air), hypoxia test (10% O_2_), and recovery conditions from 5–7 animals. In (**D**,**E**) Fulton index and hematocrit values from the four group of animals. Data represented as mean and SD from 6–10 individual data.

The effect of DOM chronic treatment is shown in Figure 2. Figure 2A shows MAP values in C rats (99 ± 23 mmHg), and the influence of chronic addition of DOM (0.75 or 1.5 mg/day) to drinking water during 2 weeks before measurements (CD; 80 ± 17 and 97 ± 15 mg Hg, respectively). In Figure 2B the effect of chronic administration of the two doses of DOM during the last 2 weeks in animals exposed to intermittent hypoxia during 30 days are represented. CIH animals had a MAP of 134 ± 32 mm Hg (** *p* < 0.01 vs. C) and the treatment with both doses of DOM during the last 2 weeks induced a MAP decreased to values of 95 ± 28 (* *p* < 0.05) and 82 ± 25 mg Hg (** *p* < 0.01) vs CIH respectively, reducing MAP to control values in CIHD group in a dose dependent manner.

In Figure 2C all blood pressure measurements are represented when animals were breathing air, a hypoxic mixture (10% O_2_/N_2_; 3 min), and recovery, breathing air again. No significant differences were observed in response to the hypoxic test among groups, using DOM (0.75 mg/day) treatment. A similar drop in MAP was observed in all animal groups breathing the hypoxic mixture, recovering previous values promptly after returning to air breathing. The percentage of hypoxia-induced MAP decrease in the four group of animals was between 52.6% ± 13.5 and 56.3% ± 7.2. Similarly, there were no statistical differences in Fulton index (Figure 2D) or hematocrit (Figure 2E) values using 0.75mg/day of DOM treatment.

### 2.2. Effects of Domperidone on CB Catecholamine Content and CB Secretory Activity 

**Figure 3:**
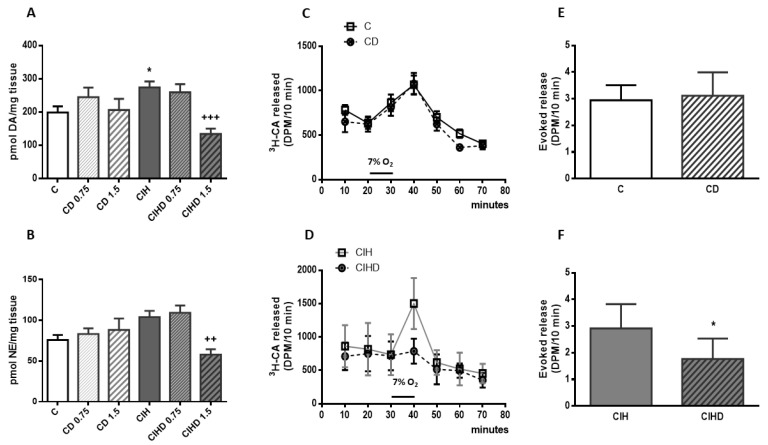
Carotid body catecholamine content and secretory activity. In (**A**,**B**) endogenous content of dopamine (DA) and norepinephrine (NE) from **C** (white bar), **C** treated with DOM (CD; 0.75 and 1.5 mg /day; 15 days; white striped bars), CIH (gray bar) and CIH treated with DOM (CIHD; 0.75 and 1.5 mg /day; 15 days; gray striped bars). Mean ± SEM from 9—18 individual data; * *p* < 0.05 vs. C; ^++^ *p* < 0.01 and ^+++^ *p* < 0.001 vs. CIH. One-way ANOVA with Tukey’s multiple comparisons test. In part (**C**), time course of ^3^H-CA secretion (DPM/10 min) elicited by 7% O_2_ from C and CD, and in part (**D**) and from CIH and CIHD animals. Data are mean and SD from 6–12 individual data. (**E**) shows the cumulative ^3^H-CA hypoxia-evoked release from C and CD and in (**F**) from CIH and CIHD group. Data are mean and SD from 6–12 individual data; *p* < 0.05 vs. CIH; unpaired t test.

In an attempt to define the participation of CB in the observed effect of DOM on arterial pressure we measured the influence of blocking D2R in CB endogenous CA content. In Figure 3 CB levels of DA and NE from the four group of animals are shown. Content of CA in CB were not significantly modified by the treatment with the two doses of DOM used (0.75 and 1.5 mg/day) in control animals, but both DA and NE content were significantly reduced in CIH rats treated with 1.5 mg/day of DOM. Figure 3A shows DA content in C (199 ± 18 pmol/mg tissue) and the increase induced by CIH (274 ± 18 pmol/mg tissue; * *p* < 0.05). C rats treated with both doses of DOM had a DA content 245 ± 28 and 206 ± 33 pmol/mg, respectively. However, the higher dose of DOM decreased DA content in CIHD group (134 ± 15 pmol/mg tissue; ^+++^ *p* < 0.001 vs. CIH) to a lower value than that of C animals. Figure 3B shows NE content in C (76 ± 6 pmol/mg) and the increase produced by CIH (104 ± 8 pmol/mg), even though it was not statistically significant. None of the doses of DOM significantly modified NE content in C animals (83 ± 7 pmol/mg and 88 ± 14 pmol/mg, respectively) but 1.5 mg/day of DOM also significantly decreased NE content in CIHD animals (58 ± 6 pmol/mg; ^++^ *p* < 0.01 vs. CIH).

It is known that hypoxia and other natural and pharmacological stimuli activate CB chemoreceptors inducing the release of CA [12,24]. Figure 3C,D show the effect of DOM treatment (0.75mg/day; 15 days) on hypoxia-evoked CA secretory response (7% O_2_) on in vitro CB from C and CIH animals. DOM did not modify ^3^H-CA secretion induced by acute hypoxic stimulus in C rats but significantly decreased it in CIH animals, as represented in parts E and F in which the accumulative ^3^H-CA hypoxia-evoked release from C and CD and from CIH and CIHD, respectively, are shown.

### 2.3. Effects of Domperidone on Respiratory Parameters

Respiratory response to acute hypoxia (12, 10, and 7% O_2_; 10 min) and hypercapnia (5% CO_2_; 10 min) tests were assessed by continuous recording of respiratory frequency (RF; breaths/min^−1^), tidal volume (TV; (mL/kg^−1^), and minute volume (MV; (ml/min^−1^/kg^−1^) in the four groups of animals. DOM (0.75 mg/day; 15 days) did not significantly modify any of the parameters in C or CIH animals. Table 1 shows RF, TV and MV in C and CD animals breathing room air (21% O_2_) and the different atmospheres. 

In room air breathing rats (basal conditions) MV was 532 ± 288 in C and 564 ± 178 ml/min^−1^/kg^−1^ in CD. Lower oxygen atmospheres increased MV reaching the maximum at 7% O_2_, the most intense hypoxic atmosphere tested in C and CD (1066 ± 201 and 1110 ± 138 ml/min^−1^/kg^−1^, respectively). MV values from CD were not significantly different compared to C animals at any hypoxia or hypercapnia stimuli tested. In every hypoxic test, CD animals shown a slight increase in MV and the opposite in the case of the hypercapnia stimulus (1151 ± 136 vs. 1041 ± 200 ml/min^−1^/kg^−1^). CIH animals showed a no significantly ventilatory response decrease at any atmosphere tested when compared to C rats. CIH reduced both, breathing frequency and/or tidal volume, producing approximately a 5–10% decrease in MV, as previously published [22,25,26]. CIH and CIHD groups breathing room air had MV values of 409 ± 82 vs. 418 ± 68 ml/min^−1^/kg^−1^, also reaching the maximum values when animals breathed 7% O_2_ (886 ± 126 vs. 953 ± 182 ml/min^−1^/kg^−1^). Values of MV in the hypercapnia test were 969 ± 97 vs. 984 ± 152 ml/min^−1^/kg^−1^ in CIH and CIHD. The decrease of MV observed in CIH was maintained in CIHD animals at every intensity of hypoxia test. Ventilatory responses in normoxia, acute hypoxia, and hypercapnia tests revealed that DOM treatment does not affect hypoxic respiratory response in C or CIH animals.

### 2.4. Effects of Domperidone on CA Content and Rate of CA Synthesis from Renal Artery (RA) 

**Figure 4:**
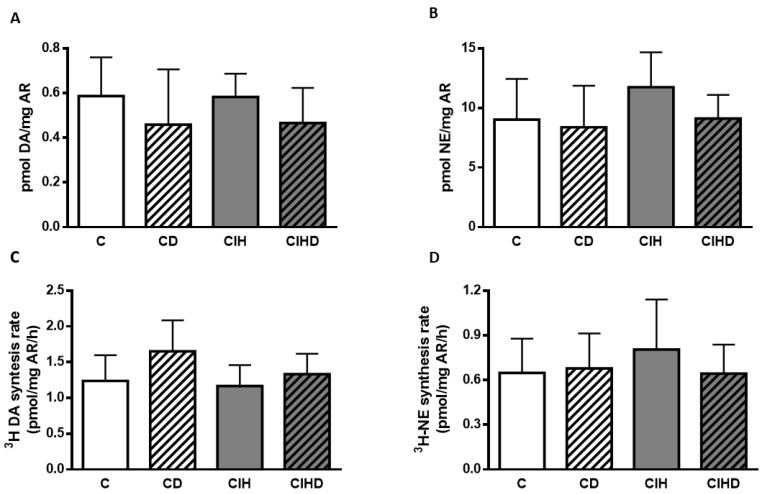
Catecholamine content and rate of synthesis from renal artery. In (**A**,**B**) content of DA and NE in renal arteries (RA) from the four group of animals. Data are mean and SD of 7–10 individual data from each group; One-way ANOVA. In (**C**,**D**) rate of ^3^H-DA and ^3^H-NE synthesis expressed as pmol/mg tissue/h in RA. Data are mean and SD from 6-10 individual values. One-way ANOVA.

To evaluate sympathetic activity, CA content and CA rate of synthesis in RA sympathetic nerve endings were measured. In control conditions (Figure 4A) content of DA in RA was 0.59 ± 0.17 pmol/mg and values were not changed by the chronic treatment with 0.75 mg/day DOM (0.46 ± 0.24 pmol/mg). Animals exposed to CIH had similar values (0.58 ± 0.10 pmol/mg) and the treatment with DOM also slightly decreased DA content (0.46 ± 0.15) pmol/mg tissue. In part B of Figure 4 content of NE in the four conditions is shown. NE content in C group was 9.01 ± 3.42 pmol/mg and the treatment with DOM gave values of 8.38 ± 3.48 pmol/mg. CIH exposure increased NE content to 11.75 ± 2.92 pmol/mg and DOM treatment lowered to 9.11 ± 1.98) pmol/mg but no statistical differences in DA or NE content were found. Figure 4C shows ^3^H-DA synthesis rate (1.24 ± 0.36 and 1.65 ± 0.43 pmol/mg RA/h) in C and CD respectively. In animals exposed to CIH, ^3^H-DA synthesis rate was 1.16 ± 0.29 pmol/mg RA/h and the treatment with DOM gave values of 1.33 ± 0.28 pmol/mg RA/h. Synthesis of ^3^H-NE is represented in part D with values of 0.65 ± 0.23 and 0.68 ± 0.23 pmol/mg/h in C and CD animals. In CIH animals ^3^H-NE synthesis rate was 0.80 ± 0.33 pmol/mg/h and DOM treatment marginally reduced it (0.64 ± 0.19 pmol/mg/h) although no statistical differences in CA synthesis rate were observed.

## 3. Discussion

Animal models have demonstrated that CIH produces CB hyperactivation followed by secretion of DA and other neurotransmitters [16]. Pre and postsynaptic D2R located in CB chemoreceptor cells and in carotid sinus nerve modulate the ventilatory response to hypoxia [27] and it is thought that DA released from CB chemoreceptor cells is regulated through feedback inhibition mediated by D2 autoreceptors [12,28]. CB is involved in the genesis of hypertension produced in CIH and the functional elimination of the chemoreceptor organ prevents hypertension from appearing [6,11,29]. In an attempt to modulate CB functionality, we sought to determine if the selective D2R blockade with DOM would reduce chemoreflex sensitivity in CIH exposed rats. The main finding was that DOM significantly lowered arterial blood pressure in CIH animals. The lowering effect on arterial pressure is observed with both acute and chronic DOM treatment and with the two doses tested. DOM marginally decreased blood pressure in control animals. The similar decrease in MAP values observed when the four group of animals breathe a hypoxic atmosphere could indicate that DOM acts, like hypoxia, decreasing vascular peripheral resistance. No differences among groups were detected in heart rate, Fulton index or hematocrit values after DOM treatment. The decrease of MAP seemed to be dose related, nearly 30% and 40% reduction with 0.75 mg/day and 1.5 mg/day of DOM chronic treatment, respectively. The acute effect of 2 mg/Kg^−1^ DOM on the hypertension induced by CIH showed a decrease of MAP of approximately 30% and no significant variation of blood pressure was observed in control animals with the same treatment. The fast effect of DOM in lowering blood pressure when administered ip could suggest a direct effect on D2R in blood vessels, which are not well described but have been found in the rat artery walls [30]. This acute effect of DOM in decreasing MAP has a possible value in treating hypertensive crisis that does not respond to standard treatment in SAHS patients. Decreasing pharmacologically the CB hypersensitivity could normalize blood pressure in SAHS patients. Our findings coincide with those of other authors who have used DOM in patients with sleep apnea disorders for the treatment of gastroesophageal reflux, observing an improvement in episodes of the apnea-hypopnea index but no measurements of arterial blood pressure were included [31]. Like other antihypertensive agents, DOM could decrease arterial pressure through sympathetic inhibition by reduction of myocardial contractility and cardiac output, vasodilation or an inhibition of plasma renin, all of which have an effect on blood pressure. In this sense, it has been reported [32] that the development of hypertension in CIH-exposed rats is due to an increase in cardiac output. The authors showed that the increased cardiac output is sufficient to explain the development of CIH-induced hypertension, which may be an early adaptive response to raise oxygen flow. Additionally, a decreased cardiac output at peak exercise using the D2R antagonist metoclopramide in healthy humans has also been showed [33], not observing changes in O_2_ consumption, CO_2_ production, or respiratory exchange ratio, highlighting the importance of endogenous DA in the normal cardiopulmonary response to exercise. DOM, a drug with therapeutic safety that has been clinically used to control gastro-esophageal motility and emesis, could be a therapeutic option for hypertension in SAHS patient’s resistant to other treatments. 

As previously reported [16,17], CB content of DA was significantly increased in CIH animals and the blocking of D2R with the higher dose of DOM reduced it below control values, even if the lower dose had no effect. These results would point to a modulating effect of the drug on the hyperstimulated CIH chemoreceptors. The treatment with DOM did not significantly modify DA content in C animals at the doses used, in which the effect of blocking D2R would eliminate the negative feedback produced by DA on D2 autoreceptors. Nevertheless, the hyperactivated CB in CIH animals would have an altered state in which DA and NE, and/or other of the numerous neurotransmitters and neuromodulators (adenosine, ATP, acetylcholine, serotonin, neuropeptides, endothelin-1, angiotensin II …), and enzymes generating gas transmitters (NO, CO, H_2_S) synthetized and released by chemoreceptor cells [12,34,35] would have modified the chemoreceptor basal condition, since chronic exposure to intermittent hypoxia deeply affects CB function. Recent evidence suggests that dysregulation of transcriptional activators, HIF-1 and HIF-2 and the consequent variance in oxidant enzyme genes leads to raised ROS production [36] that could disturb neurotransmitter signaling in CB. In this study, 1.5 mg/day during 2 weeks of DOM treatment reduced the increased CA in CB from CIH animals, not altering these values in control conditions. These results would indicate that DOM withdraws a tonic inhibitory control on the CIH hyperactivated chemoreflex. However, the lower dose did not significantly modify CA content in CB, although it produced a decrease in blood pressure which could be due to CB independent mechanisms. The stimulus-evoked release response supported this modulating effect of DOM by significantly decreasing the CA release response to hypoxic stimulation in CIH rats. However, the use of DOM to limit or counteract the dopaminergic neuromodulation of CB chemosensitivity did not have effect on respiratory parameters. DOM produced a slight but not significant increase or no change in MV in both C and CIH animals when they breathed air or the different hypoxia or hypercapnia tests. These results would agree with those [37] studying DOM effect on ventilation in chronic hypoxic rats, in which they observed a stimulating effect on the ventilatory response to hypoxia when acute DOM was administered, but after 2 weeks of DOM treatment no significant differences were observed. It was also found [38] that DOM did not affect the hypoxic ventilatory response of newborn intermittent hypoxic rats although it increased baseline ventilation. Other authors [39] did not observe a relationship between plasma DOM concentration and the degree of augmentation of the hypoxic response in humans. Differences with acute DOM treatment in breathing frequency and MV have been found depending upon rat genetic background [40,41]. It has been also described [42] that acute DOM administration significantly increased MV in rats breathing hyperoxia but not hypoxia atmospheres. The discrepancy of results obtained with DOM can be related to strain differences and/or due to the difference between acute or chronic administration of the drug. Furthermore, basal condition of CB chemoreceptors (control, chronic hypoxia or intermittent hypoxia) would alter D2R dynamics in CB. This is the first report of chronic DOM treatment (2 weeks) in adult CIH rats and could be not compared to the previously published results obtained from animals exposed to chronic sustained hypoxia, acute DOM treatment, or effects of DOM on newborn animals. 

It is currently accepted that the CB is the origin of the sympathetic hyperactivity observed in chronic exposure to intermittent hypoxia in animal models [22,26,43,44,45] and enhanced peripheral chemoreflex sensitivity and sympathetic activity is also marked in SAHS patients [46,47,48]. The sympathetic nervous system is also related to sleep initiation and disruption. When disruptions become chronic, autonomic impairment may follow, leading to increased sympathetic drive [49]. DA receptors have been studied mostly in central nervous system but their peripheral role is not well known. Circulatory DA levels come mainly from spilling over from noradrenergic nerves [50] and even low concentration of DA is reported to be sufficient to activate DA receptors in vascular smooth muscle cells, in a paracrine or autocrine manner. In kidney, DA is synthetized and secreted from proximal tubule cells decreasing sodium transport, and the alteration of DA signaling is implied in some types of hypertension [51,52,53]. D2R mainly signal through Gi protein alpha subunit, which inhibit adenyl cyclase, Ca^2+^ and K^+^ channels, and consequently produce vasoconstriction. Additionally, DA is produced and released by the vascular endothelium and hypoxia stimulates CA release in those cells [30,54]. Renal artery content and synthesis rate of CA were measured as an index of sympathetic activity. DA content in RA was not significantly modified by CIH or DOM treatment. Similarly, no significant differences were observed in NE content, although the slight increase in content and synthesis rate of NE would indicate that renal sympathetic activity is augmented in CIH animals, suggesting a role in the genesis of hypertension in animals exposed to CIH in which DOM treatment would lead CA to control values, but data are not conclusive. Endogenous content of CA from RA has a partial value in relation to the actual state of activity of the sympathetic nervous system, so although we have not found a significant decrease of CA content after treatment with DOM, it cannot be ruled out that an effect has occurred. Acute hypoxia has been reported to increase myocardial NE turnover in rats, but chronically increased sympathetic activity is usually associated with no change or a decrease in tissue CA levels [6]. Results presented a marginal increase in renal sympathetic activity in CIH animals that could influence the increased blood pressure and that the effect of DOM would be the reduction of CA levels. It has been also reported [55] the lack of effect of DOM on renal hemodynamics or sodium excretion in rats. CIH in animal models or in SAHS patients augments sympathetic discharges in different sympathetic nerve areas [29] and it has been described that chemoreflex mediated sympathetic activation also contributes to intermittent hypoxia-induced hypertension by enhancing CA secretion from the adrenal medulla [56,57]. The effect of DOM acting through the adrenal medulla cannot be ruled out. The study has not conclusively demonstrated a causal relationship between the effects of DOM on CB (and RA) catecholamine content and secretion and the hypotensive effects in CIH-exposed rats so that new experiments will be needed. Limitations of the study: (i) physiological recording of the CB afferent sensory nerve activity (carotid sinus nerve) in CIH and C animals with and without DOM would allow us to elucidate the implication of CB as a target of DOM effects; additionally, to study the CB redox status and modifications on ROS production in the different experimental conditions would also help to clarify the CB implication. (ii) measuring renal artery nerve activity, along with plasma CA levels and CA content in sympathetic tissues would inform about DOM action on modifying CIH sympathetic overactivation. (iii) other cardiovascular targets should also be considered. 

In summary, this study demonstrated that DOM can reverse the hypertension induced by chronic exposure to intermittent hypoxia in rats, and the CB could be involved in this modulation through the blocking of D2R. The underlying mechanisms implicated in the effect of DOM on lowering blood pressure in the CIH rat model need further investigation.

## 4. Materials and Methods 

Experiments were carried out in compliance with the applicable international laws and policies (European Union Directive for Protection of Vertebrates Used for Experimental and Other Scientific Ends (2010/63/EU), and were reviewed and approved by the University of Valladolid Institutional Committee for Animal Care and Use (Project Approval Ethical Code: 4505502, 14th, Oct, 2019).

### 4.1. Animals and Anesthesia 

Young adult male Wistar rats were used, with free access to standard food and water and maintained under controlled conditions of temperature, humidity and a stationary light–dark cycle. Rats were randomly distributed in four different groups: Control (C); control with DOM administered in the drinking water at doses of 1.5 or 0.75 mg/day during the last 15 days before the experiments (CD); chronic exposure to intermittent hypoxia (cycles of 40 s, 5% O2/80 s, air; 8 h/day) during 30 days as described previously [22] (CIH); and CIH during 30 days with DOM administered in the drinking water (1.5 or 0.75 mg/day) during the last 15 days of intermittent hypoxia exposure (CIHD). Weight and drinking behavior were not different among the four groups of animals. To assess the acute effect of DOM another set of animals were used, divided into two groups, C and CIH during 15 days. Except for CIH exposure and whole body plethysmography, experimental procedures were performed in animals anaesthetized with sodium pentobarbital (60 mg/kg^−1^ body weight; i.p.) or ketamine plus diazepam (100 and 1.6 mg/kg^−1^, respectively; i.p.). At the end of experiments, animals were euthanized by the administration of a lethal dose of sodium pentobarbital. 

### 4.2. Arterial Blood Pressure Measurement 

Arterial blood pressure and heart rate were recorded from ketamine 100 mg/Kg-1 and diazepam 2 mg/Kg^−1^; i.p. anesthetized animals, tracheostomized and ventilated with room air (CL Palmer) (60 cycles. min-1 and a positive end-expiratory pressure of 2 cm H_2_O) or with the hypoxia gas mixture (10% O_2_ and 90% N_2_; 3 min). Arterial blood pressure was continuously monitored by a catheter located in the right common carotid artery and connected to a pressure transducer (Transpac IV; ICU Medical, San Clemente, CA, USA). Signals were stored (BIOPAC Systems, Inc. MP 150, Goleta, CA, USA; Acknowledge 3.9.1) for later analysis. After arterial blood pressure measurements were completed, a 0.2 ml arterial blood sample was collected for hematocrit determination. After rats were euthanized, heart dissection was performed and right (RV) and left ventricle plus septum (LV+S) were weighed to calculate the Fulton Index (RV/LV+S). 

### 4.3. Plethysmography 

Respiratory parameters as tidal volume (TV; mL/Kg), respiratory frequency (RF; breaths/min), and minute ventilation (MV; mL/min/Kg) were obtained by whole-body, unrestrained plethysmography. Methacrylate-walled chambers (Emka Technologies, Paris, France; Buxco Research Systems, Wilmington, NC, USA) continuously fluxed (1.5 L/min) with air, hypoxic gas mixtures (12, 10, and 7% O_2_, reminder nitrogen) and hypercapnic gas mixture (5% CO_2_ in air) were used as previously described [17,26]. Animals breathed air until achieving a standard resting behavior inside the chambers; temperature was maintained within the thermo-neutral range (22–24 °C). Pressure modifications inside the chamber reflecting TV were calculated with a high-gain differential pressure transducer. Amplitude of pressure fluctuations is proportionally correlated to TV; a calibration of the system by injections of 5 mL air into the chamber allowed a direct estimation of TV. All parameters were recorded and analyzed with FinePointe software (Buxco Research Systems).

### 4.4. Endogenous Catecholamine Content

Endogenous catecholamine (CA) content, in CB and renal artery (RA), were analyzed after removing the tissues from anesthetized animals, glass to glass homogenized (0.1N perchloric acid (PCA) and 0.1 mM EDTA), centrifuged and processed for HPLC-ED analysis as described before [17].

### 4.5. Stimuli–Evoked Catecholamine Release from CB and Renal Artery Catecholamine Synthesis 

Isolated CB were incubated 2 hours with 3,5-3H-tyrosine (30 µM) of high specific activity (40–50 Ci/mmol; Perkin Elmer, Boston, MA, USA) and cofactors for tyrosine hydroxylase and dopamine beta hydroxylase, 100 μM 6-methyl-tetrahydropterine and 1 mM ascorbic acid, respectively, to evaluate stimulus-evoked secretory response. Later, CB were moved to vials with Tyrode-bicarbonate solution (in mM: 116 NaCl, 5 KCl, 2 CaCl_2_, 1.1 MgCl_2_, 10 HEPES, 5 glucose, 24 NaCO_3_H) equilibrated with gas mixtures containing 21 or 7% O_2_ and 5% CO_2_ (pH 7.40). Incubating solutions were changed every 10 min to measure 3H-CA content by scintillation counter as previously described [58]. RA were incubated (37 °C; 2h) in Tyrode solution, containing 30 μM of 3,5-3H-tyrosine (6 Ci/mmol), as described above; then, isolated RA were washed in precursor-free Tyrode solution (4 °C; 5min), homogenized (0.1N PCA and 0.1 mM EDTA), centrifuged and processed for HPLC-ED analysis. General procedures have been described before [26]. 

### 4.6. Data Presentation and Statistical Analysis 

Results are presented as mean and SD. Statistical analyses were performed by GraphPad Prism version 6.0 (GraphPad Software, San Diego, CA, USA). Mean value comparisons were made using unpaired Student’s t-test, One-Way Analysis of Variance (ANOVA) or Two-way ANOVA with Tukey’s or Sidak’s multi-comparison test, according to the structure of data, considering a p-value < 0.05 as statistically significant. 

## Figures and Tables

**Table 1:** Respiratory parameters in control and chronic intermittent hypoxia animals and the effect of chronic domperidone treatment. The table shows breathing frequency, tidal volume and minute volume from the four group of animals breathing at different atmospheres. Data are mean and SD of eight individual values in each group. In the upper part control (C) and control with DOM (CD; 15 days with 0.75 mg/day of DOM treatment). In the lower part the same respiratory parameters from animals exposed to intermittent hypoxia during 30 days (CIH) and CIH treated with DOM (CIHD; 0.75 mg/day during 15 days). Two-way ANOVA showed no statistical differences among groups.

**Animals**	**C**			**CD**		
**Respiratory** **Parameter**	**Breathing Frequency** **(Breaths min^−1^)**	**Tidal Volume** **(mL kg^−1^)**	**Minute Ventilation** **(mL min^−1^ kg^−1^)**	**Breathing Frequency** **(Breaths min^−1^)**	**Tidal Volume** **(mL kg^−1^)**	**Minute Ventilation** **(mL min^−1^ kg^−1^)**
21% O_2_ (air)	129 (93)	4.6 (0.5)	532 (288)	114 (47)	5.4 (0.5)	564 (178)
12% O_2_	131 (23)	5.4 (0.7)	664 (45)	160 (17)	5.7 (0.7)	809 (178)
10% O_2_	139 (30)	5.1 (0,7)	921 (100)	134 (26)	6.2 (0.6)	951 (89)
7% O_2_	139 (30)	8.0 (0.8)	1066 (201)	134 (26)	8.6 (0.8)	1110 (138)
5% CO_2_ in air	150 (24)	7.9 (0.8)	1151 (136)	141 (30)	7.6 (0.8)	1041 (200)
**Animals**	**CIH**			**CIHD**		
**Respiratory** **Parameter**	**Breathing Frequency** **(Breaths min^−1^)**	**Tidal Volume** **(mL kg^−1^)**	**Minute Ventilation** **(mL min^−1^ kg^−1^)**	**Breathing Frequency** **(Breaths min^−1^)**	**Tidal Volume** **(mL kg^−1^)**	**Minute Ventilation** **(mL min^−1^ kg^−1^)**
21% O_2_ (air)	92 (31)	4.9 (0.5)	409 (82)	93 (28)	5.0 (0.9)	418 (68)
12% O_2_	124 (17)	5.3 (0.7)	622 (74)	119 (17)	5.4 (0.7)	624 (106)
10% O_2_	152 (28)	6.1 (0.8)	851 (156)	147 (16)	6.4 (1.0)	892 (81)
7% O_2_	121 (12)	7,6 (1.1)	886 (126)	123 (18)	7.9 (1.1)	953 (182)
5% CO_2_ in air	130 (66)	5.2 (0.6)	969 (97)	132 (17)	7.6 (1.0)	984 (152)

## References

[1] Konecny T., Kara T., Somers V.K. (2014). Obstructive sleep apnea and hypertension: an update. Hypertension.

[2] Fletcher E.C., Lesske J., Qian W., Miller C.C., Unger T. (1992). Repetitive, episodic hypoxia causes diurnal elevation of blood pressure in rats. Hypertension.

[3] Chen L., Zhang J., Gan T.X., Chen Izu Y., Hasday J.D., Karmazyn M., Balke C.W., Scharf S.M. (2008). Left ventricular dysfunction and associated cellular injury in rats exposed to chronic intermittent hypoxia. J. Appl. Physiol..

[4] Del Rio R., Andrade D.C., Lucero C., Arias P., Iturriaga R. (2016). Carotid Body Ablation Abrogates Hypertension and Autonomic Alterations Induced by Intermittent Hypoxia in Rats. Hypertension.

[5] Mcnicholas W.T., Bonsigore M.R. (2007). Management committee of EU cost action B26. Sleep apno ea as an independent risk factor for cardiovascular disease: current evidence, basic mechanisms and research priorities. Eur. Respir. J..

[6] Fletcher E.C., Lesske J., Culman J., Miller C.C., Unger T. (1992). Sympathetic denervation blocks blood pressure elevation in episodic hypoxia. Hypertension.

[7] Peng Y.J., Yuan G., Khan S., Nanduri J., Makarenko V.V., Reddy V.D., Vasavda C., Kumar G.K., Semenza G.L., Prabhakar N.P. (2014). Regulation of hypoxia-inducible factor-α isoforms and redox state by carotid body neural activity in rats. J. Physiol..

[8] Narkiewicz K., Ratcliffe L.E., Hart E.C., Briant L.J., Chrostowska M., Wolf J., Szyndler A., Hering D., Abdala A.P., Manghat N. (2016). Unilateral carotid body resection in resistant hypertension: a safety and feasibility trial. JACC Basic. Transl. Sci..

[9] Fujii K., Saku K., Kishi T., Oga Y., Tohyama T., Nishikawa T., Sakamoto T., Ikeda M., Ide T., Tsutsui H. (2017). Carotid Body Denervation Markedly Improves Survival in Rats with Hypertensive Heart Failure. Am. J. Hypertens..

[10] Iturriaga R. (2018). Carotid Body Ablation: A New Target to Address Central Autonomic Dysfunction. Curr. Hypertens Rep..

[11] Lesske J., Fletcher E.C., Bao G., Unger T. (1997). Hypertension caused by chronic intermittent hypoxia--influence of chemoreceptors and sympathetic nervous system. J. Hypertens..

[12] Gonzalez C., Almaraz L., Obeso A., Rigual R. (1994). Carotid body chemoreceptors: from natural stimuli to sensory discharges. Physiol. Rev..

[13] Gauda E.B. (2002). Gene expression in peripheral arterial chemoreceptors. Microsc. Res. Tech..

[14] Wakai J., Takayama A., Yokoyama T., Nakamuta N., Kusakabe T., Yamamoto Y. (2015). Immunohistochemical localization of dopamine D2 receptor in the rat carotid body. Acta Histochem..

[15] Ford C.P. (2014). The role of D2-autoreceptors in regulating dopamine neuron activity and transmission. Neuroscience.

[16] Hui A.S., Striet J.B., Gudelsky G., Soukhova G.K., Gozal E., Beitner-Johnson D., Guo S.-Z., Sachleben L.R., Haycock J.W., Gozal D. (2003). Regulation of catecholamines by sustained and intermittent hypoxia in neuroendocrine cells and sympathetic neurons. Hypertension.

[17] Docio I., Olea E., Prieto-LLoret J., Gallego-Martin T., Obeso A., Gomez-Niño A., Rocher A. (2018). Guinea Pig as a Model to Study the Carotid Body Mediated Chronic Intermittent Hypoxia Effects. Front. Physiol..

[18] Gonzalez-Obeso E., Docio I., Olea E., Cogolludo A., Obeso A., Rocher A., Gomez-Niño A. (2017). Guinea Pig Oxygen-Sensing and Carotid Body Functional Properties. Front. Physiol..

[19] Laduron P.M., Leysen J.E. (1979). Domperidone, a specific in vitro dopamine antagonist, devoid of in vivo central dopaminergic activity. Biochem. Pharmacol..

[20] Kohli J.D., Glock D., Goldberg L.I. (1983). Selective DA2 versus DA1 antagonist activity of domperidone in the periphery. Eur. J. Pharm..

[21] Schey R., Saadi M., Midani D., Roberts A.C., Parupalli R., Parkman H.P. (2016). Domperidone to Treat Symptoms of Gastroparesis: Benefits and Side Effects from a Large Single-Center Cohort. Dig. Dis. Sci..

[22] Quintero M., Olea E., Conde S.V., Obeso A., Gallego-Martin T., Gonzalez C., Montserrat J.M., Gomez-Niño A., Yubero S., Agapito M.T. (2016). Age protects from harmful effects produced by chronic intermittent hypoxia. J. Physiol..

[23] Perrim R.R., Bonagamba L.G., Machado B.H. (2015). Cardiovascular and respiratory outcome of preconditioned rats submitted to chronic intermittent hypoxia. Exp. Physiol..

[24] Chen J., Gomez-Nino A., Gonzalez C., Dinger B., Fidone S. (1997). Stimulus-specific mobilization of dopamine and norepinephrine stores in cat carotid body. J. Auton. Nerv. Syst..

[25] Gonzalez-Martín M.C., Vega-Agapito M.V., Conde S.V., Castaneda J., Bustamante R., Olea E., Perez-Vizcaino F., Gonzalez C., Obeso A. (2011). Carotid body function and ventilatory responses in intermittent hypoxia. Evidence for anomalous brainstem integration of arterial chemoreceptor input. J. Cell. Physiol..

[26] Olea E., Agapito M.T., Gallego-Martin T., Rocher A., Gomez-Niño A., Obeso A., Gonzalez C., Yubero S. (2014). Intermittent hypoxia and diet-induced obesity: effects on oxidative status, sympathetic tone, plasma glucose and insulin levels, and arterial pressure. J. Appl. Physiol. (1985).

[27] Carroll J.L., Boyle K.M., Wasicko M.J., Sterni L.M. (2005). Dopamine D2 receptor modulation of carotid body type 1 cell intracellular calcium in developing rats. Am. J. Physiol. Lung Cell Mol. Physiol..

[28] Iturriaga R., Larraín C., Zapata P. (1994). Effects of dopaminergic blockade upon carotid chemosensory activity and its hypoxia-induced excitation. Brain Res..

[29] Prabhakar N.R., Peng Y.J., Jacono F.J., Kumar G.K., Dick T.E. (2005). Cardiovascular alterations by chronic intermittent hypoxia: importance of carotid body chemoreflexes. Clin. Exp. Pharmacol. Physiol..

[30] Bucolo C., Leggio G.M., Drago F., Salomone S. (2019). Dopamine outside the brain: The eye, cardiovascular system and endocrine pancreas. Pharmacol. Ther..

[31] Larrain A., Kapur V.K., Gooley T.A., Pope C.E. (2010). Pharmacological treatment of obstructive sleep apnea with a combination of pseudoephedrine and domperidone. J. Clin. Sleep Med..

[32] Lucking E.F., O’Halloran K.D., Jones J.F. (2014). Increased cardiac output contributes to the development of chronic intermittent hypoxia-induced hypertension. Exp. Physiol..

[33] Tedjasaputra V., Bryan T.L., Van Diepen S., Moore L.E., Bouwsema M.M., Welsh R.C., Petersen S.R., Stickland M.K. (2015). Dopamine receptor blockade improves pulmonary gas exchange but decreases exercise performance in healthy humans. J. Physiol..

[34] Kumar P., Prabhakar N.R. (2012). Peripheral chemoreceptors: function and plasticity of the carotid body. Compr. Physiol..

[35] Nurse C.A. (2014). Synaptic and paracrine mechanisms at carotid body arterial chemoreceptors. J. Physiol..

[36] Prabhakar N.R., Semenza G.L. (2012). Adaptive and maladaptive cardiorespiratory responses to continuous and intermittent hypoxia mediated by hypoxia-inducible factors 1 and 2. Physiol. Rev..

[37] Gamboa J.L., Macarlupú J.L., Rivera-Chira M., Monge C.C., León-Velarde F. (2003). Effect of domperidone on ventilation and polycythemia after 5 weeks of chronic hypoxia in rats. Respir. Physiol. Neurobiol..

[38] Julien C.A., Joseph V., Bairam A. (2011). Alteration of carotid body chemoreflexes after neonatal intermittent hypoxia and caffeine treatment in rat pups. Respir. Physio. Neurobiol..

[39] Walsh T.S., Foo I.T., Drummond G.B., Warren P.M. (1998). Influence of dose of domperidone on the acute ventilatory response to hypoxia in humans. Br. J. Anaesth..

[40] Subramanian S., Dostal J., Erokwu B., Han F., Dick T.E., Strohl K.P. (2007). Domperidone and ventilatory behavior: Sprague–Dawley versus Brown Norway rats. Respir. Physiol. Neurobiol..

[41] Nakano H., Lee S.D., Farkas G.A. (2002). Dopaminergic modulation of ventilation in obese Zucker rats. J. Appl. Physiol. (1985).

[42] Huey K.A., Brown I.P., Jordan M.C., Powell F.L. (2000). Changes in dopamine D(2)-receptor modulation of the hypoxic ventilatory response with chronic hypoxia. Respir. Physiol..

[43] Prabhakar N.R., Kumar G.K. (2010). Mechanisms of sympathetic activation and blood pressure elevation by intermittent hypoxia. Respir. Physiol. Neurobiol..

[44] Xing T., Pilowsky P.M., Fong A.Y. (2014). Mechanism of sympathetic activation and blood pressure elevation in humans and animals following acute intermittent hypoxia. Prog. Brain Res..

[45] Iturriaga R., Oyarce M.P., Dias A.C.R. (2017). Role of Carotid Body in Intermittent Hypoxia-Related Hypertension. Curr. Hypertens Rep..

[46] Narkiewicz K., Somers V.K. (2003). Sympathetic nerve activity in obstructive sleep apnoea. Acta Physiol. Scand..

[47] Beecroft J., Duffin J., Pierratos A., Chan C.T., McFarlane P., Hanly P.J. (2006). Enhanced chemo-responsiveness in patients with sleep apnoea and end-stage renal disease. Eur. Respir. J..

[48] Tamisier T., Tan C.O., Pepin J.L., Levy P., Taylor J.A. (2015). Blood Pressure Increases in OSA du5 to Maintained Neurovascular Sympathetic Transduction: Impact of CPAP. Sleep.

[49] Miglis M.G. (2016). Autonomic dysfunction in primary sleep disorders. Sleep Med..

[50] Goldstein D.S., Holmes C. (2008). Neuronal source of plasma dopamine. Clin. Chem..

[51] Carey R.M. (2001). Theodore Cooper Lecture: Renal dopamine system: paracrine regulator of sodium homeostasis and blood pressure. Hypertension.

[52] Gildea J.J., Shah I., Weiss R., Casscells N.D., McGrash H.E., Zhang J., Jones J.E., Felder R.E. (2010). HK-2 human renal proximal tubule cells as a model for G protein-coupled receptor kinase type 4-mediated dopamine 1 receptor uncoupling. Hypertension.

[53] Armando I., Villar V.A., Jose P.A. (2015). Genomics and Pharmacogenomics of Salt-sensitive Hypertension. Curr. Hypertens Rev..

[54] Sorriento D., Santulli G., Del Giudice C., Anastasio A., Trimarco B., Iaccarino G. (2012). Endothelial Cells Are Able to Synthesize and Release Catecholamines both In Vitro and In Vivo. Hypertension.

[55] Jose P.A., Asico L.D., Eisner G.M., Pocchiari F., Semeraro C., Felder R.A. (1998). Effects of costimulation of dopamine D1- and D2-like receptors on renal function. Am. J. Physiol..

[56] Bao G., Metreveli N., Li R., Taylor A., Fletcher E.C. (1997). Blood pressure response to chronic episodic hypoxia: Role of the sympathetic nervous system. J. Appl. Physiol..

[57] Semenza G.L., Prabhakar N.R. (2018). The role of hypoxia-inducible factors in carotid body (patho) physiology. J. Physiol..

[58] Rocher A., Caceres A.I., Almaraz L., Gonzalez C. (2009). EPAC signalling pathways are involved in low P-O2 chemoreception in carotid body chemoreceptor cells. J. Physiol..

